# Estimation of the impact of Fasciola hepatica infection on time taken for UK beef cattle to reach slaughter weight

**DOI:** 10.1038/s41598-017-07396-1

**Published:** 2017-08-04

**Authors:** Stella Mazeri, Gustaf Rydevik, Ian Handel, Barend M. deC. Bronsvoort, Neil Sargison

**Affiliations:** University of Edinburgh, Royal (Dick) School of Veterinary Studies and The Roslin Institute, Easter Bush Veterinary Centre, Roslin, Midlothian, EH26 0PE UK

## Abstract

Fasciolosis is common in UK beef cattle, but it is unclear at what levels liver fluke burdens cause production losses. This study aimed to address these uncertainties by estimating the impact of liver fluke infection on UK beef cattle productivity and investigating the use of diagnostic tests in a quantitative manner. We built three linear regression models for slaughter age by weight and different measures of liver fluke status, while accounting for sex, breed, season, year and farm of origin. Data were sourced from Scotland’s largest red meat abattoir throughout 2013 and 2014. Our Meat Hygiene Service model estimated that cattle classified as having liver fluke damage had on average 10 days greater slaughter age than animals with no evidence of fasciolosis. Our liver fibrosis model estimated that the increase in slaughter age was more severe for higher fibrosis scores. Similarly, our burden model showed an increase in slaughter age for animals with as few as 1 to 10 parasites found in their livers. Lastly, we used receiver operating characteristic curves to show that serum antibody ELISA, copro-antigen ELISA and faecal egg counting can be useful in distinguishing between animals with and without production limiting levels of fasciolosis.

## Introduction

Parasites of the genus *Fasciola* are of worldwide importance, causing disease in multiple mammalian species including humans^1^. In the British Isles, fasciolosis caused by *Fasciola hepatica* ^2^ is a major production limiting disease of ruminant livestock. Fasciolosis is claimed to cost the UK cattle industry £23 million annually^3^, a figure that remains a crude estimate as the true effect on production is unclear. There has been an increase in the incidence of fasciolosis and geographical spread of the problem in the British Isles in the last decade; a trend that has been related to global climate change and extensive animal movements^4, 5^. This trend has been predicted to continue in the coming decades throughout Europe^6, 7^.

Cattle are less susceptible to showing clinical signs of fasciolosis compared to small ruminants, with a higher infection challenge of metacercariae required to cause clinical disease^8, 9^. This is thought to be due to the large size of the liver, which leads to a greater functional reserve, and that the liver has a more fibrous texture than in other animals^9^. Fasciolosis in cattle therefore mainly manifests as a subclinical chronic disease, associated with hepatic damage and blood loss caused by parasites in the bile ducts^10^. There are indications that cattle might develop partial immunity with age (unlike e.g. sheep)^11, 12^. At the same time, the risk of infection appears to increase with age, supporting the hypothesis that immunity does not prevent re-establishment of new infection^4^.

While subclinical infections cause reduced production levels and may contribute to pasture contamination, it is difficult to convince farmers to invest in the control of fasciolosis without demonstrating the economic cost of subclinical disease^11^. In fact, the lack of obvious clinical signs of fasciolosis in cattle results in these losses commonly being attributed to other causes such as poor weather conditions or undernutrition. To date, it has been difficult to estimate the effects of subclinical infection due to a lack of quantitative production and parasite burden data^13^.

There was a number of studies on the impact of liver fluke infection on production carried out in the 70 s to 90 s, which presented conflicting results^8^. While a few studies failed to show a relationship^14, 15^, several studies showed that liver fluke infection was associated with a range of production level effects^11, 16, 17^. A more recent study in Louisiana USA^18^, reported a 6% body weight increase in crossbred heifers treated for liver fluke compared to an untreated group. However, since these studies involved experimentally infected animals of limited types or breeds, their relevance to naturally infected cattle populations is unclear. Similarly, it is difficult to assume that these results would apply to the modern day production of beef cattle.

Two recent abattoir-based studies have attempted to address this. In one study of 1450 Belgian Blue suckler cows, sourced from 480 herds slaughtered at the abattoir of Velzeke in Belgium, Charlier *et al*.^19^, investigated the relationship between *F. hepatica*-specific meat juice antibody ELISA results with carcass parameters including carcass weight, conformation score and fat coverage using linear and logistic regression models. The results showed that an increase over the interquartile range of the mean herd serum antibody ELISA results was associated with a 3.4 kg lower mean herd carcass weight. Nevertheless, they did not identify significant associations between *F. hepatica*-specific meat juice antibody ELISA titres and carcass characteristics, at the individual animal level. In a second study, Sanchez-Vazquez *et al*.^20^ analysed data collected between 2005 and 2010 from 328,137 cattle of various beef breeds slaughtered at a large beef abattoir in Aberdeenshire, Scotland. Using regression modeling and adjusting for factors such as for an animal’s age, breed and sex, season, year and farm, they showed that liver fluke infected cattle had on average 0.63 kg lower carcass weights and lower carcass conformation scores than non-infected animals^20^.

The impact of parasitic infections on production depends upon the severity of challenge, the duration of exposure, the effect on metabolism, host immunity, and the metabolic cost of a competent immune system^21^, and if this is true for *Fasciola*, we would expect to see a relationship between severity of disease and production losses. In addition to confirming the results of Sanchez-Vazquez *et al*.^20^, our study therefore includes two measures of disease progression that have not previously been used in the literature: fibrosis scores, and fluke burden, in addition to using condemned livers. Furthermore, our study complements the work by Charlier *et al*.^19^, by assessing the ability of diagnostic tests used in the live animal of identifying animals with production limiting levels of infection. Our study analyses data collected over a two-year period on 169,605 prime cattle slaughtered at Scotland’s largest red meat abattoir and:

- Estimates the difference in slaughter age, corrected for weight, between beef cattle infected with liver fluke vs. uninfected cattle using meat inspection results as an indication of infection status.
- Estimates the difference in slaughter age, corrected for weight, of beef cattle with different liver fibrosis scores used as an indication of the extent of liver fluke infection.
- Estimates the difference in slaughter age, corrected for weight, of beef cattle with different levels of parasite burden as an indication of the extent of infection.
- Investigates the quantitative use of diagnostic tests in distinguishing between animals of high and low levels of infection.

### Descriptive results

#### Routine abattoir data

**Figure 1:**
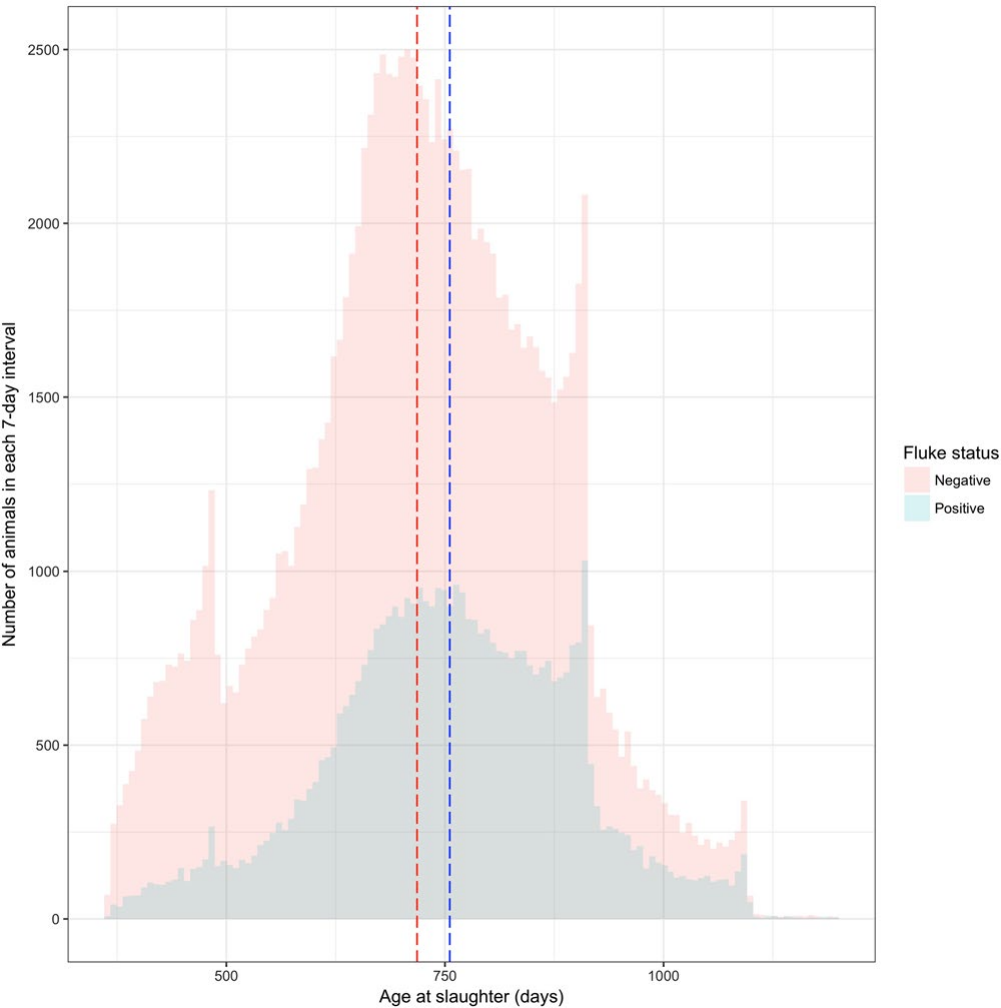
Distribution of age of cattle at slaughter by liver fluke status. This figure compares the age distribution at the time of slaughter between animals who had livers rejected due to signs of liver fluke infection and those who did not using data from the two year abattoir dataset (n = 169,605). Dotted lines show the mean ages at which fluke negative (red) and fluke positive (blue) cattle are slaughtered. There is a 37 days difference between the two means.

**Figure 2:**
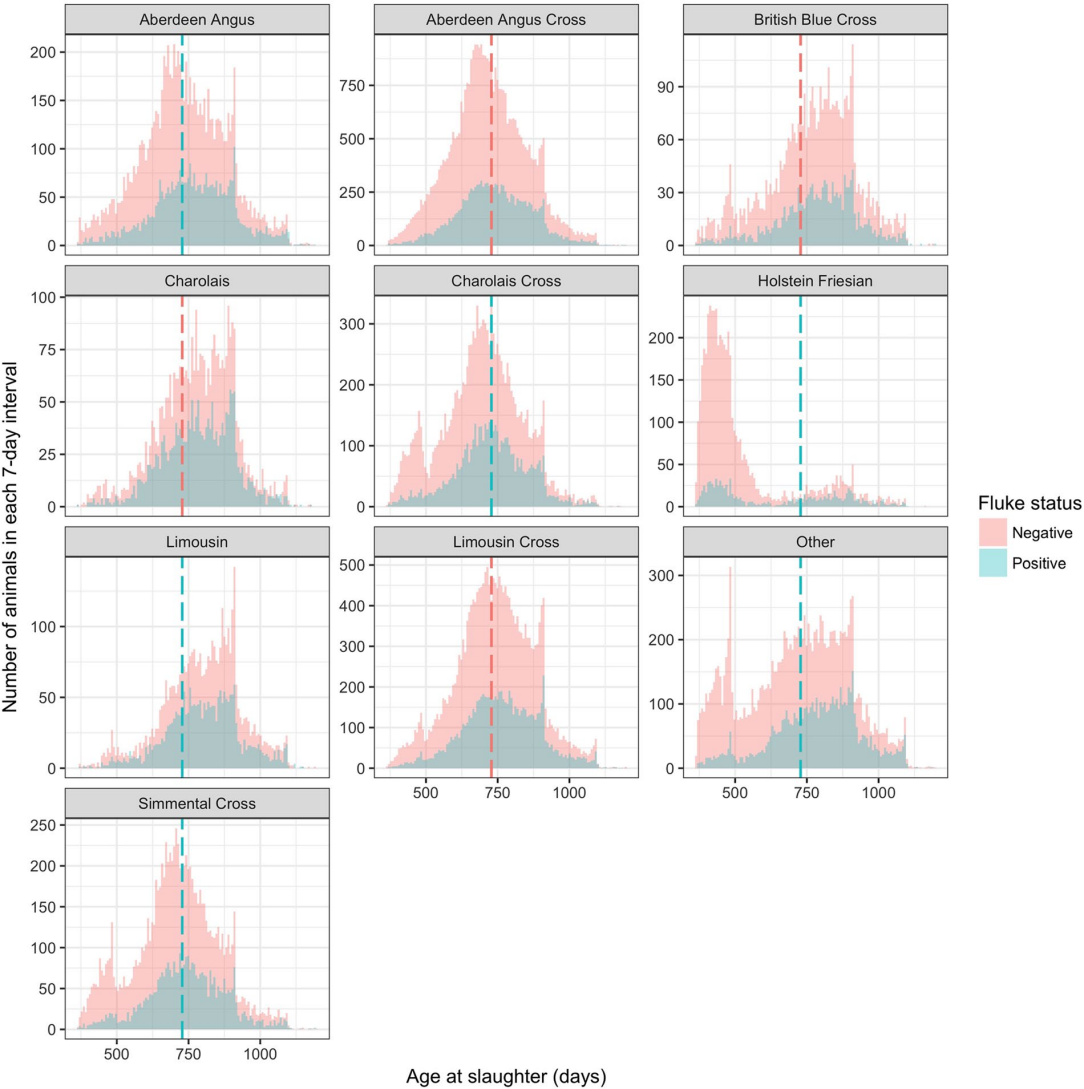
Distribution of age of cattle at slaughter by breed and liver fluke status. This figure compares the age distribution at the time of slaughter for different breeds between animals who had livers rejected due to signs of liver fluke infection and those who did not using data from the two year abattoir dataset (n = 169,605). Dotted lines show the mean ages at which fluke negative (red) and fluke positive (blue) cattle are slaughtered. The difference in age of positive and negative animals varies between different breeds, but the mean age of fluke positive animals remains greater or equal to fluke negative animals of all breeds.

A large dataset containing information about 169,605 cattle was made available and used for the Meat Hygiene Service (MHS) model. The dataset included all cattle breeds slaughtered at the abattoir, while excluding cull cows. The age of the animals at the time of slaughter ranged from 366 to 1,199 days. Information on sex was available for 59,321 female and 103,423 male cattle. The cattle were sourced from 1,724 different producers that varied greatly in the number of slaughtered cattle, ranging from 1 to 8332 animals, with a median of 27 animals per producer. Overall, 45,452 cattle (28%) had livers rejected by the MHS service due to signs of liver fluke infection. Figures 1 and 2 show the age distribution of these animals according to liver fluke status based on the results of the liver inspection by the MHS alone and by breed. The mean age at slaughter was found to be greater for animals with livers rejected due to liver fluke when compared to animals without liver rejection, irrespective of the breed of the animal. Table 1 shows the different breeds of cattle according to whether or not they had livers rejected due to signs of liver fluke.

**Table 1:** Distribution of animals with livers rejected due to liver fluke, across the different breeds and across the sex of the animals.

Variable	Levels	No of livers rejected	Percentage rejected	Total
BREED	Aberdeen Angus	3,477	27.6%	12,602
	Aberdeen Angus Cross	12,901	24.8%	52,032
	British Blue Cross	1,452	26.0%	5,577
	Charolais	1,906	35.7%	5,332
	Charolais Cross	5,123	28.1%	18,201
	Holstein Friesian	1,018	16.4%	6,197
	Limousin	2,152	35.2%	6,105
	Limousin Cross	8,408	27.7%	30,359
	Other	5,419	27.4%	19,781
	Simmental Cross	3,596	26.8%	13,419
SEX	Female	16,458	27.7%	59,321
	Male	26,814	25.9%	103,423

#### Abattoir based sampling

**Figure 3:**
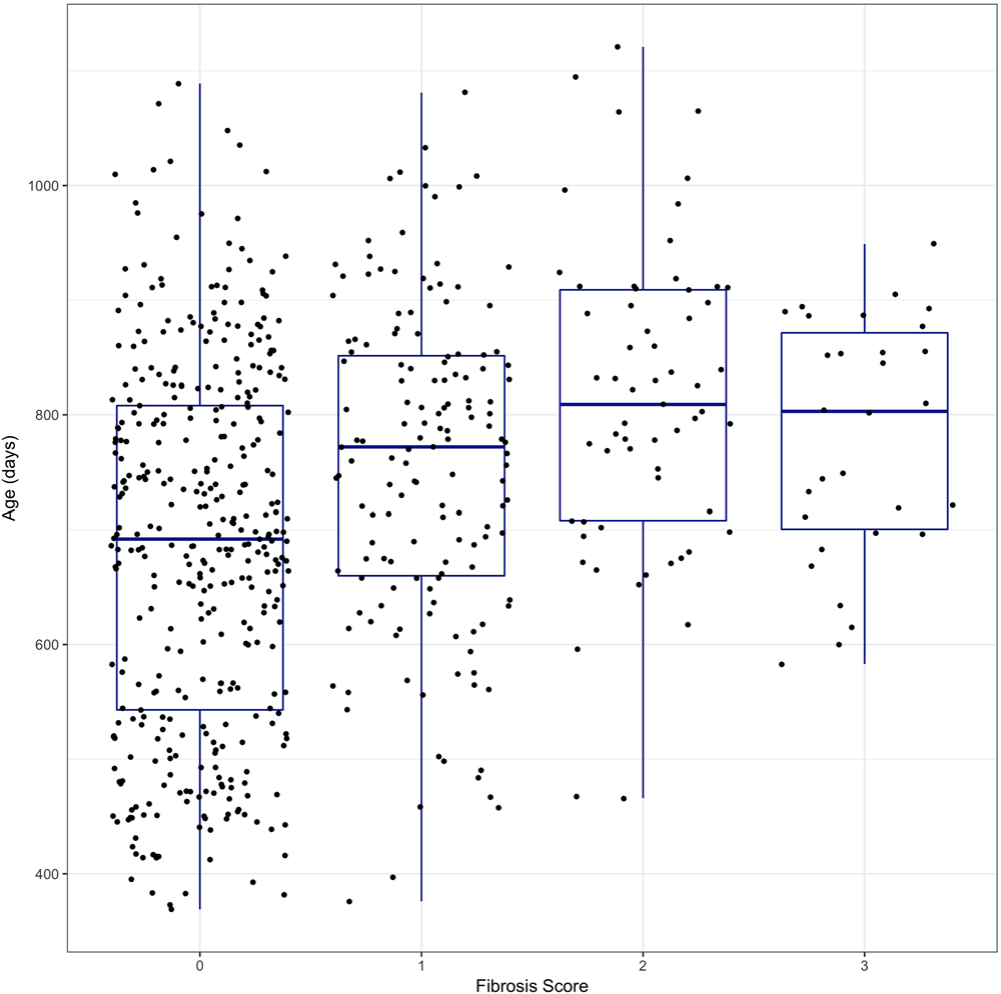
Distribution of cattle age at slaughter by fibrosis score. Figure shows box plots and actual values of cattle age at slaughter for each fibrosis score (n = 619). There is a positive age trend as fibrosis score increases from 0 to 3, but there is great variability of age values for each score.

**Figure 4:**
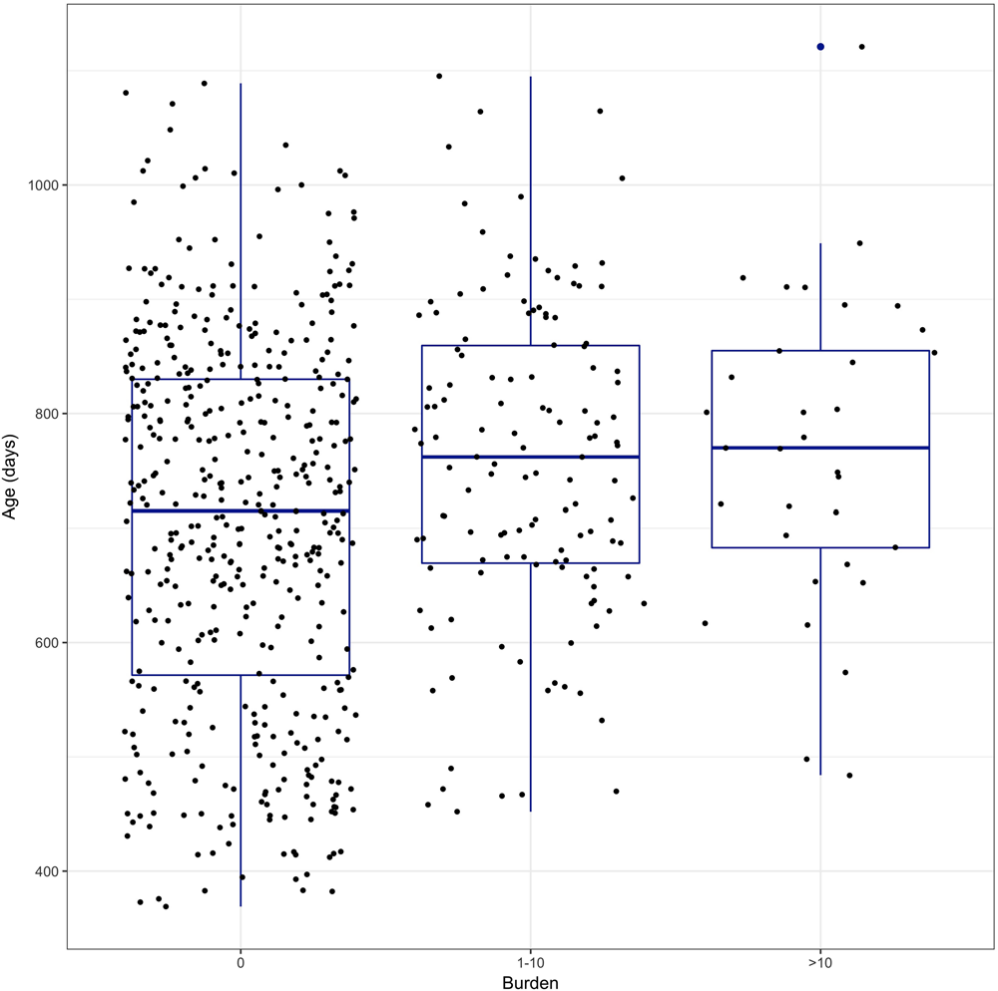
Distribution of cattle age at slaughter by burden category. Figure shows box plots and actual values of cattle age at slaughter for each burden category (n = 619). Slaughter age is higher in animals with a burden of more than 0, but the slaughter age distributions of animals of burden 1–10 and >10 are very similar.

**Figure 5:**
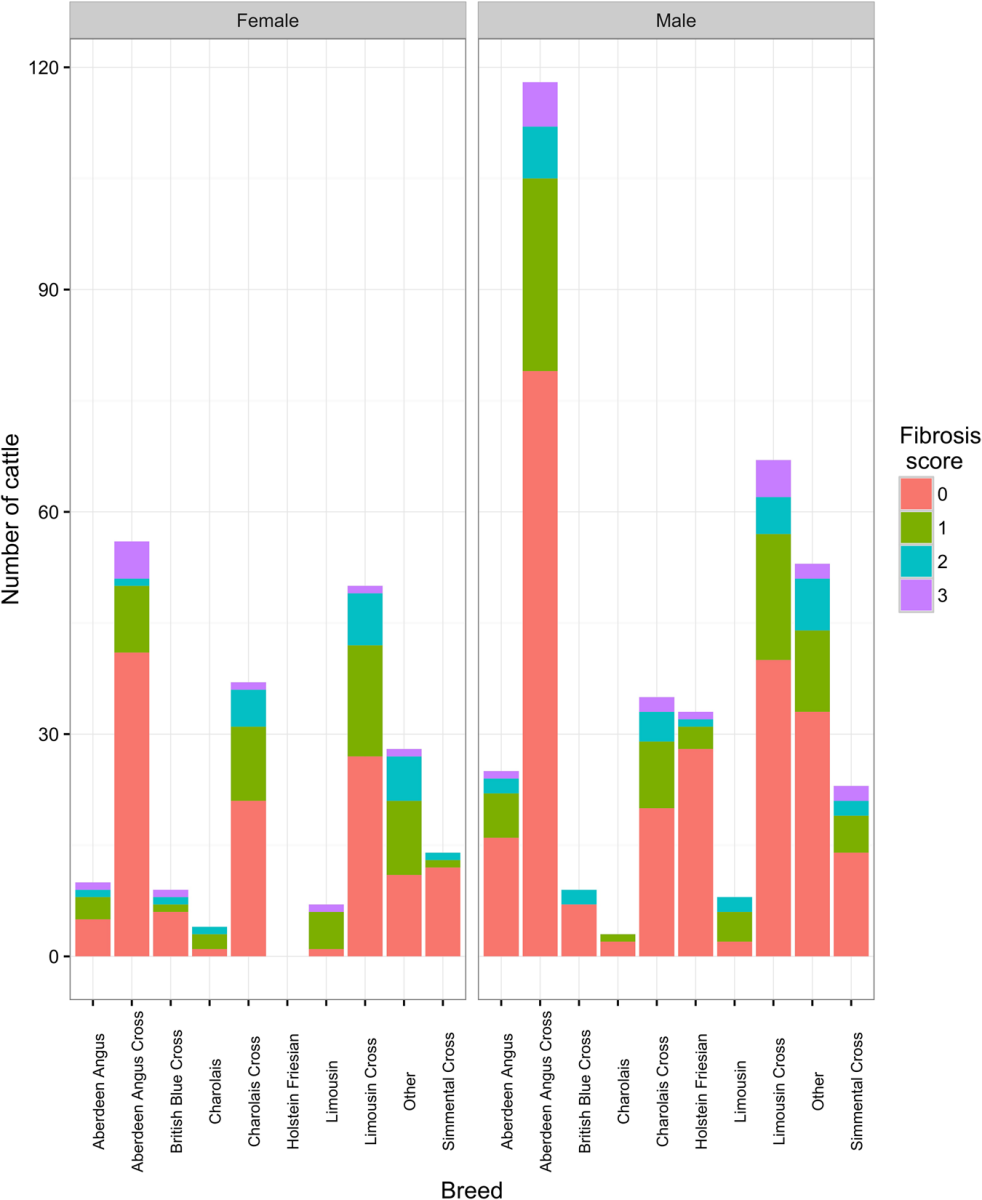
Distribution of fibrosis scores by cattle breed and sex. Figure shows the distribution of fibrosis scores according to sex and breed of sampled animals.

619 cattle were sampled during the three sampling periods sourced from 255 different producers. Sampled cattle had a slaughter age range of 369 to 1,121 days. One or more parasites were identified in 164 of the 619 animals sampled (burden distribution shown in Supplementary Fig. S1). Figures 3 and 4 show the slaughter age distribution of these animals according to their fibrosis score and level of burden respectively. Sex data were available for 589 of sampled animals of which 215 were female and 374 were male. Figure 5 shows the distribution of fibrosis scores among the various breeds and sex of sampled animals (exact figures can be found in Supplementary Table S1).

### Regression modeling

The first analysis used liver inspection results from the full 2013 to 2014 abattoir dataset as an indicator of infection, and fitted a linear mixed model to the relationship between slaughter age and liver fluke condemnation and other covariates (for full details, see the methods section). The results indicated that there was a significant relationship between the presence of liver fluke infection and the slaughter age, when accounting for sex, breed, age, and farm-level effects. More precisely,an animal with a mean slaughter carcass weight of 345 kg took on average 10 (95% 9–12) days longer to reach slaugher weight if it had its liver rejected due to liver fluke compared to animals who did not have livers rejected. The estimates of all model parameters are shown in Supplementary Table S2.

As seen in figure two, the mean observed age difference between animals with different liver statuses was similar across breed with the exception of Holstein-Friesian (HF) cattle, where animals with rejected livers were substantially older than for other breeds. We therefore conducted two subgroup analyses: one sub-analysis excluding HF cattle, and one sub-analysis including only observations from HF cattle. The results from the first sub-analysis showed little change, estimating a mean delay of 9 days (95% CI 8–11 days) for cattle other than HF. The second sub-analysis indicated that HF cattle with rejected livers took on average 23 days (95% CI 15–31 days) longer to finish than those where the livers were not rejected.

**Figure 6:**
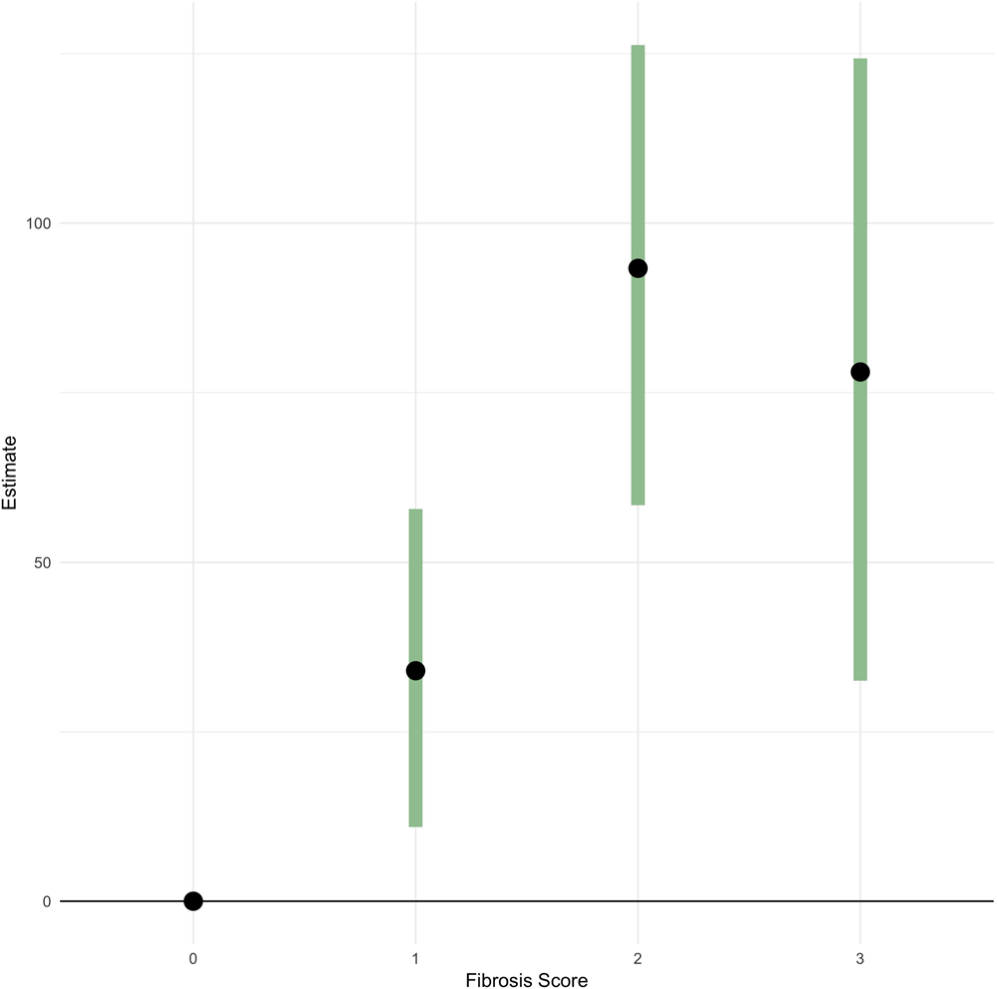
Point estimates and 95% Confidence Intervals for different fibrosis scores. Figure shows the estimated increase in slaughter age in days for an animal with 345 kg carcass weight for fibrosis scores 1–3 compared to a fibrosis score of 0, as estimated by the fibrosis model.

We then used fibrosis score as an indicator of severity on disease, using the subset of 619 cattle that had been sampled for detailed diagnostics. Fitting a similar statistical model as above, the result showed that animals with a mean slaughter carcass weight of 345 kg and a fibrosis score of 1 (147 animals) was linked to an additional 34 (95% CI 11–57) days to finish when compared to cattle with fibrosis score 0 (381 animals). This effect was more pronounced for animals with more severe disease - animals with a fibrosis score 2 (61 animals) took 93 days (95% CI 58–127 days) longer to finish; and animals with a fibrosis score 3 (30 animals) took 78 days (95% CI 31–125 days) longer to finish (Fig. 6).

**Figure 7:**
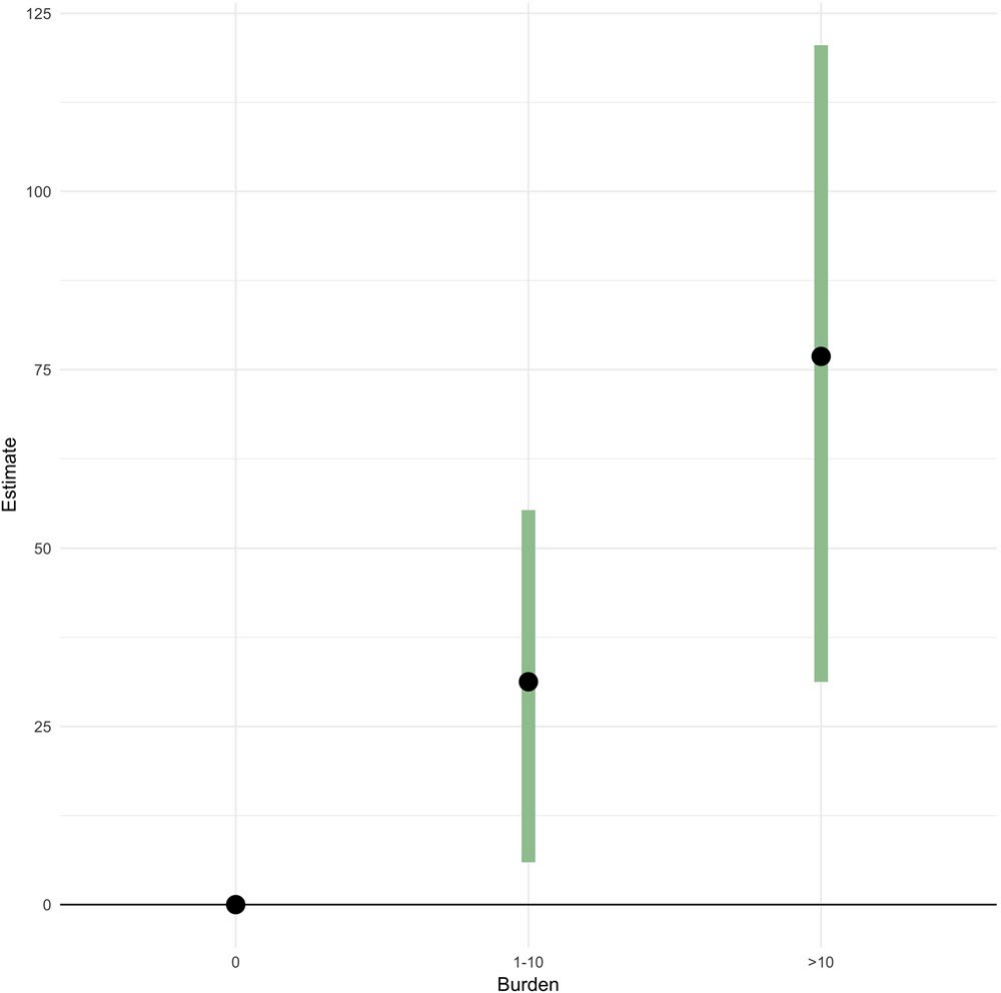
Point estimates and 95% Confidence Intervals for animals with different levels of burden compared to animals with 0 burden. Figure shows the estimated increase in slaughter age in days for an animal with 345 kg carcass weight for burden categories 1–10 and >10 compared to a burden of 0, as estimated by the burden model.

The final analysis used parasite burden as an indicator of disease, categorised into animals with 0 parasites, 1–10 parasites, and more than ten parasites. The results from the model indicated that animals with a mean slaughter carcass weight of 345 kg and a parasite burden of 1–10 liver flukes (131 animals) took 31 days (95% CI 7–55 days) longer to finish when compared to cattle with no parasites found in their livers (455 animals). Correspondingly, animals with a parasite burden of more than 10 liver flukes (33 animals) took 77 days (95% CI 31–122 days) longer to finish (Fig. 7). Detailed estimates of model parameters for the second and third models can be found in Supplementary Tables S3 and S4 respectively.

### Relationship between diagnostic tests and fluke infection

**Figure 8:**
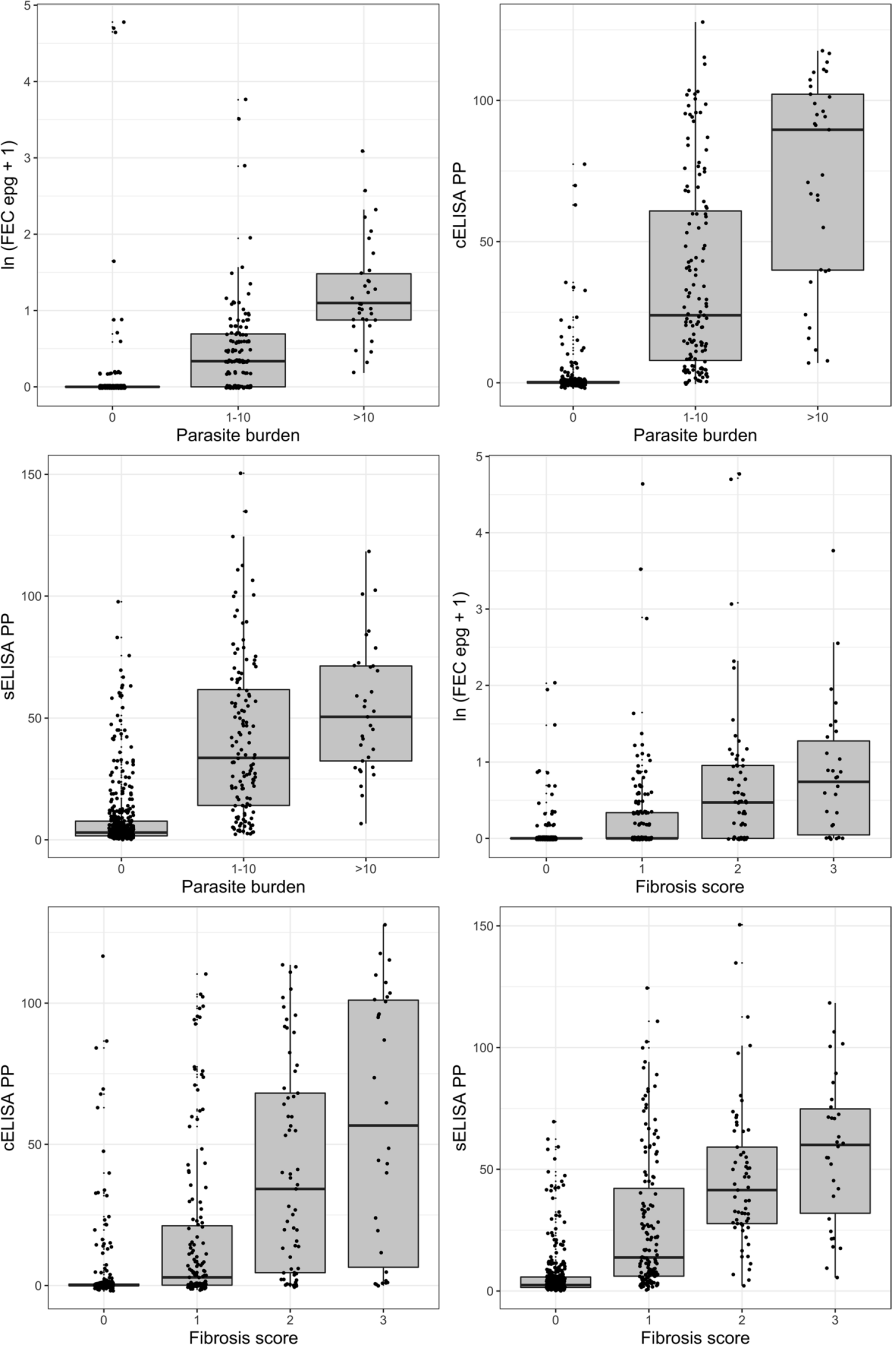
Relationship between diagnostic tests and severity of disease. Plots show the raw results of each diagnostic test vs. parasite burden (left) and fibrosis score (right). There are positive trends between test results and increasing parasite burden and fibrosis scores, but there is great variability in actual values.

The number of animals tested and the proportion of animals classified as positive for each of the three *F. hepatica* laboratory diagnostic tests are shown in Table 2. Continuous results of the three tests against parasite burden and fibrosis scores are shown in Fig. 8. While the results are clearly variable, we see a positive trend for all three tests when comparing quantitative tests results to either parasite burden or fibrosis score. This trend is further supported by the highly significant Spearman rank correlation coefficients between test results and parasite burden and fibrosis score shown in Table 3 (p < 0.001 for all coefficients).

**Table 2:** Number of animals tested and the measured prevalence for each of the four types of diagnostic tests used.

Diagnostic test	Total tested	Number positive	Prevalence
Liver Necropsy	619	196	0.32
FEC	619	143	0.23
cELISA	619	148	0.24
sELISA	619	223	0.36

**Table 3:** Spearman rank correlation coefficients between the number of parasites in the liver, fibrosis score and continuous test results of each diagnostic test. The spearman rank correlation is a nonparametric test of whether there is a statistical relationship between two variables. A correlation coefficient of 1 indicates a perfect positive correlation, while a correlation coefficient of −1 indicates a perfect negative correlation and a correlation coefficient of 0 indicates no correlation. In this case p < 0.001 for all coefficients indicate that these correlations were statistically significant.

Diagnostic Test	Liver fluke burden	Fibrosis score
FEC eggps per gram	0.79	0.57
cELISA PP	0.72	0.54
sEISA PP	0.62	0.67

The diagnostic tests we used (FEC, sELISA, and cELISA) have recommended cut-off values for identifying animals with fasciolosis, but these cut-offs are not necessarily optimal for identifying animals with production-relevant levels of disease. In order to evaluate the practical ability of each test to distinguish animals with high burden of disease, we therefore used Receiver operating characteristic (ROC) curves analysis to identify test cut-off values that provide optimal combination of sensitivity and specificity relative to high fibrosis score and burden measures. Based on the results of our fibrosis model, which indicate that animals with fibrosis scores of 2 or more are likely to have a greater slaughter age than animals with milder fibrosis, it was considered useful to see how well diagnostic tests distinguish between animals with fibrosis scores of two or more and animals with no pathology or fibrosis score of 1. Similarly, we chose a fluke burden of more than 10 parasites, a cut-off suggested as an economic threshold in previous work^22^, as a measure of high fluke burden.

**Figure 9:**
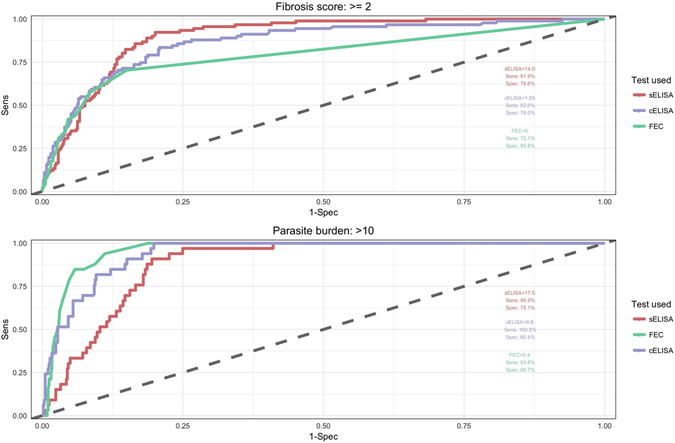
ROC curves. ROC curves were used to evaluate the ability of each diagnostic test to distinguish between 1) animals with a fibrosis score of 2 or more vs. a fibrosis score of 0 or 1 (top plot), and 2) animals with a parasite burden of more than 10 vs. animals with 10 or fewer flukes (bottom plot). Each plot provides suggested cut-off values for each tests along with the sensitivity and specificity estimates of each test at that cut-off value.

ROC curves of the 3 diagnostic tests for differentiating between animals with different fibrosis scores and parasite burdens are shown in Fig. 9. At the suggested cut-offs reported on the plot (Fig. 9, top), the sELISA and the cELISA have high sensitivities for detecting animals with fibrosis scores 2 or higher (91.9% and 82.6% respectively), and moderate specificities of 79.6 and 79.0%, respectively. FECs have a relatively low sensitivity of 72.1% at detecting animals with fibrosis scores of 2 or more, while the specificity is slightly higher than that of the other tests (84.8%).

The lower graph in Fig. 9 shows the ROC curves for using the tests to detect animals with more than ten liver flukes. All three tests - sELISA, cELISA, as well as FEC - have high sensitivity at (96.9%, 100.0% and 93.8%, respectively) for detecting high-burden animals at the suggested cutoffs. FECs also have a high specificity of 88.7%, while the sELISA and cELISA have lower specificities (75.1% and 80.4%, respectively).

## Discussion

The results presented in this paper provides further evidence that infection with liver fluke in cattle is associated with a substantial delay to reach slaughter weight when compared to uninfected cattle. We have estimated that a typical beef animal yielding a 345 kg carcass takes on average 10 days longer to reach slaughter weight (adjusted for breed, sex, and farm-level effects) if evidence of fasciolosis is reported by the MHS, than if it is not. Our results are consistent with the impact of fasciolosis that was shown in the only other published large scale UK abattoir study of liver fluke infection in beef cattle^20^, which estimated that fluke infection was associated with on average 0.63 kg lower slaughter weight reduction, controlling for age.

The results from these two abattoir studies are in line with earlier studies reporting a negative effect of fluke on growth rates^11, 16, 23^. However, most of these studies were based on experimentally infected cattle, representing small numbers of animals, and different breeds, or management systems^22^, and the relevance of their results in a production setting is unclear. It has therefore historically been difficult to demonstrate an economic impact of fasciolosis in cattle, without which it is difficult for producers to justify investment in disease control. Estimating the financial impact of fasciolosis on beef production is also increasingly important to allow UK beef cattle producers to evaluate control measures in the face of a changing prevalence of *F. hepatica* ^9, 24^. Together with Sanchez-Vazques *et al*.^20^, the results in this paper strengthen the evidence for the economic impact of fasciolosis on beef production under present-day conditions.

As described in results section, a subgroup analysis indicated that for Holstein Friesian cattle, the estimated difference in slaughter age was on average 23 days between animals with and without condemned livers, compared to 10 days for all animals. However, Holstein Friesian cattle differed in a number of other ways, in particular by having a much lower overall slaughter age compared to the other breeds. The difference in estimated delay to slaughter can therefore be due to a number of different factors, including the average age at slaughter, and the different management of dairy breeds compared to beef breeds. The analysis results with and without Holstein Friesian cattle were very similar, which is expected as we are already controlling for breed and slaughter age in the regression. However, the interaction between fasciolosis epidemiology, farming systems, and heterogenous demographics should be considered and examined in future studies.

There is limited development of immunity to liver fluke; hence older animals have a higher prevalence of fasciolosis, which confounds weight and age at slaughter as indicators of the effect of infection on growth rate. Older animals with more recent infections also risks masking potential negative effect of long-term disease. Finally it is possible that the observed delay in reaching slaughter weight is (in some part) due to reverse causality - that weaker or more slow-growing animals are more likely to be infected with liver fluke. In order to address these concerns, we used a second dataset of 619 animals sampled over three different periods, to investigate the effects of liver fluke according to severity of fibrosis and parasite burden. Our fibrosis and burden models show that slaughter age increases as severity of infection increases. More specifically, our fibrosis model shows that a typical beef animal yielding a 345 kg carcass and having a liver fibrosis score of 1, 2, or 3 takes on average 34, 93, and 78 days longer, respectively, to reach slaughter weight when compared to animals with no liver fibrosis detected. Our burden model likewise shows that when compared with animals with no liver fluke burden, animals with 1 to 10 parasites take on average 31 days longer to reach slaughter weight, while animals with more than 10 *F. hepatica* flukes in their liver at slaughter take 77 days longer to finish. According to basic epidemiological principles^25^, a dose-response relationship between exposure and effect provides further evidence for a causal relationship between the two. While the results from the fibrosis model do not show a strict dose-response relationship (though note that the effect estimates for highest category are based on relatively few animals and have wide confidence intervals), combined with the results from the burden model, they provide some additional evidence that increased levels of liver fluke are related to substantially lower beef cattle growth rates in a production setting. More importantly, they provide an indication of the potential scale of the production effects of severe disease. Our results therefore highlight the importance of knowing the extent of infection, rather than solely determining whether or not an animal is infected.

Previous reports have implied that significant production losses only occur when liver *F. hepatica* burdens exceed 30 flukes^26^ or 10 flukes^22^. However, the burden model presented in our study suggests that animals with as few as 1 to 10 parasites identified in their livers after slaughter grow at a slower rate than uninfected animals. The conflicting results highlight the complexity of the relationship between levels of liver fluke infection identified at slaughter and production loss, which is dependent on factors such as the duration of infection, previous treatments, feed quality and animal housing management^26^.

The MHS classification of liver condemnation is a valuable part of the abattoir health surveillance, but it has limitations. Liver inspection by the MHS has been estimated to have a sensitivity of 68% and a specificity of 88%^27^, thereby misclassifying 32% of truly positive animals and 12% of truly negative animals. When using the MHS results as a response variable, this will result in regression dilution bias^28^, leading to an underestimation of, for example, the effect of liver fluke infection on slaughter age. By using more specific measures of infection, such as fibrosis score, this effect can be reduced. For example, our fibrosis and burden models both estimate a greater delay in reaching slaughter weight than our MHS model, at all levels of fibrosis and burden. It should be noted that while fasciolosis is the most common reason for bovine liver fibrosis seen in abattoirs, other causes of bovine liver fibrosis include *Dicrocoelium dendriticum* infections, pyrrolizidine alkaloid and other plant toxicoses as well as mineral toxicosis (copper, iron, zinc). Certain clostridial diseases can also be involved, but these are often a result of liver compromise due to fasciolosis^29, 30^. Because of this, any measure of fibrosis will have some limitations when used as an indicator of fasciolosis. However, the conclusions from this study regarding the level of production loss in beef cattle due to fasciolosis is strengthened by the use of three different models; the MHS model, the stronger results of the fibrosis model, and the fact that these are consistent with the results of the burden model.

Tests for the diagnosis of liver fluke infection in cattle have historically been used solely in a qualitative manner with the aim of identifying whether an animal is infected or not, but giving no indication of the extent of infection. However, owing to chronic and predominantly sub-clinical nature of fasciolosis in cattle, producers require information about the intensity of infection. Knowledge of how this relates to production loss is needed to instigate preventive management strategies, involving host evasion of high levels of metacercarial challenge, grazing management to reduce snail and free-living fluke stage habitats, and the strategic use of flukicidal drugs with the aim of interrupting the parasite life cycle through reduced host egg shedding^24^. This requires the quantitative use of diagnostic tests to identify when infected animals actually require treatment and to inform producers about the extend of production loss they are suffering due to liver fluke infection^31^. In this context, we investigated the potential of three available diagnostic tests (FEC, cELISA and sELISA) to be used quantitatively. Positive trends were identified for the relationship between the results of all three tests and measures of the extent of infection, both graphically and using Spearman rank correlation coefficients (Table 3). The correlation of FECs and parasite burden is generally considered weak^32^, although our study shows that there is a strong positive correlation. We also found a good correlation between cELISA results and parasite burden, supporting the results of previous studies^22, 33, 34^. However, the correlations between FEC and cELISA results and fibrosis scores were found to be weak in our study. Reports on the correlation between sELISAs and parasite burden range from no correlation^35–37^, to positive correlation^38–40^. Our study shows a weak positive correlation of sELISA results with fluke burden and a moderate positive correlation with fibrosis score. In summary, test result values at each level of *F. hepatica* burden and fibrosis appear to be variable, making it difficult to directly quantify actual levels of infection or damage based on continuous test results.

However, by considering them as regular binary tests but changing the cut-off values, the tests can still be adapted to better identify animals with severe disease that may be of production relevance. We used ROC curves to evaluate how well the tests can distinguish between cattle with high and low fluke burdens^22^ and between different levels of liver damage based on the results of our fibrosis model. In our study, when using the cut-offs identified by ROC analysis regarding high parasite burden, all three tests have high sensitivity in detecting animals with more than 10 liver flukes. FECs have a high specificity, while the cELISA and sELISA tests have lower specificities. These results indicate a potential for using the tests in practice to inform strategies on reducing both production loss and pasture contamination, by providing a way of identifying which animals are more likely to be infected by a greater number of parasites and hence adjusting treatment strategies accordingly. Similarly, when using the cut-offs identified by ROC analysis regarding liver fibrosis, the cELISA and sELISA tests appear more sensitive in picking up animals that have suffered greater damage, while having moderate specificities. In this context, tests can be used to inform producers on the extent of the damage caused by fasciolosis, providing a means of monitoring and adjusting control strategies accordingly. Depending on the situation, i.e. whether the user is interested in measuring damage already suffered by the animal or the current parasitic burden, one could use the cut-off suggested for each test in terms of fibrosis or burden respectively.

The results presented in this paper highlight the value of abattoir data in veterinary clinical research. The availability of these data combined with results of abattoir based sampling, has enabled us to build robust models to investigate the impact of liver fluke infection on beef cattle production in a representative naturally-infected population representative of the UK beef cattle population. We have presented a simple fibrosis scoring system which can be carried out within the same time frame as routine liver inspection, providing producers with more informative feedback on the extent of fasciolosis in their herds, allowing the iterative implementation of more effective and sustainable control strategies.

This type of abattoir study has proven to be able to provide a range of results of relevance to beef production, but it has certain unavoidable limitations. When using abattoir data for the estimation of the effect of diseases on production, weight is commonly used as an outcome variable. In this paper, we chose to use age at slaughter. If weight is used as an outcome variable, it is assumed that the weight can be explained by the predictive variables, and importantly, that any variability is additive to the sum of these predictive variables. However, the weight at which an animal is sent for slaughter is more or less predetermined by the farms’ targets combined with market prices. Variability seen in slaughter weight will be mainly due to breed and sex differences of animals, originating from systems with different target weights. Thus, effects of liver fluke infection on production will have little impact on slaughter weights. In other words, the error structure is different from that assumed by using weight as a response, and therefore confidence intervals (and p-values) will be misleading, and effects of fluke likely underestimated.

Regardless of whether weight or age is used as a response, there is an additional epidemiological issue. Fluke prevalence increases with age, because of accumulated exposure to the parasite - in our dataset, prevalence increases linearly from 13% for animals slaughtered at 300 days to 40% for animals slaughtered at 1100 days. However, farmers will delay sending animals that are slow-growing in order to reach desired target weights. If fluke infection affects growth, then we would therefore expect fluke-infected animals to arrive to slaughter later than non-infected animals. Thus, fluke might affect growth, which affects age until the animal is sent to slaughter, which in turn affects fluke prevalence. This type of potential cycle of causality is recognised as simultaneity bias in the literature^41–43^, and will make it difficult to separate out prevalence effects from growth effects. Preliminary simulation analyses indicate that even in the case of no effect of fluke on growth, there would be a high probability of getting significant results from this type of data from a regression analysis.

Given the evidence provided by this and other studies of the potential importance of fluke to beef production, it would be valuable to conduct further studies that are better able to identify the relationship between fluke infection and the health and growth of cattle, and disentangle the causal web. If limited to abattoir data, there are examples in the literature of methods that might be able to account for simultaneity bias (e.g the instrumental variable approach), if additional variables fulfilling certain criteria can be collected^44, 45^. However, we would suggest that a stronger study design would be a prospective longitudinal study following up animals from birth to slaughter, recording growth rates and presence of fluke infection at set intervals, hence providing further evidence on the relationship between fluke infection and growth rates in a naturally infected cattle population. Based on the results of previous work by the authors^27^ and further insight in diagnostics tests presented in this paper, the test of preference for this kind of study is likely to be cELISA, as it has been shown to perform equally well during different seasons (cf FEC), has greater specificity than the sELISA^27^ and can give some idea of burden. The most conclusive results would be generated by an intervention study whereby one would randomize farms and/or animals to three arms; no treatment, blanket treatment and selective treatment, i.e. using diagnostic tests to selectively treat animals with high burden, and relate that to growth rate data of animals on each arm of the study.

Overall, our study provides robust evidence that cattle infected with liver flukes take substantially longer to reach slaughter weight compared with non-infected animals, and that this impact depends on the extent of infection. Additionally, we have provided further evidence to correlate diagnostic test results with measures of infection or morbidity to enable the identification of animals with high burdens or high degrees of fibrosis, and hence improve the efficiency of control strategies. Finally, we suggest a fibrosis score methodology to be routinely used at slaughterhouses in order to provide more informative liver inspection results.

## Methods

### Data sources

#### Data routinely collected at the abattoir

Data used in this study were sourced from Scotbeef Limited, the largest red meat abattoir in Scotland. The dataset included information that is routinely collected by the Meat Hygiene Service (MHS) about animals that were slaughtered at Scotbeef abattoir between 3rd January 2013 and 10th November 2014, such as reasons given for offal or carcass rejection following inspection, along with carcass characteristics such as weight and grade. It is mandatory for livers of all cattle slaughtered in European abattoirs to be inspected (EC Regulation No 854/2004), and livers identified with signs of infection upon visual inspection, palpation or incision of the gastric surface of the liver must be condemned^46^. For the purposes of this study results of liver inspection by the MHS were used as an indication of liver fluke infection.

#### Abattoir based sampling

The second dataset used in this study is a combination of data collected using abattoir based sampling and the two year dataset described above. A detailed description of the sampling strategy can be found in Mazeri *et al*.^27^. Briefly, blood, faeces, whole livers and gall bladder samples were collected from each animal during three sampling periods during June and July 2013, January and March 2014, and August and October 2014. During each period, samples were collected over six sampling days, once per week, sampling 32 to 36 animals per day. Samples were collected systematically from one in every 10 cattle slaughtered. This was to ensure that the samples were representative of animals slaughtered during the whole day, and for logistical reasons to allow time for processing between samples.

#### Cattle Tracing System (CTS) database

All cattle in the United Kingdom are identified by their unique eartag number and have a passport issued by the British Cattle Movement System (BCMS) containing information on animal specific details of sex, breed, date of birth and death and any movements that occurred throughout the animal’s life^47^. This information is held within the BCMS Cattle Tracing System (CTS) database^48^. We collected each sampled animal’s unique eartag number, which for the purposes of this study enabled us to obtain data on its farm of origin, age, breed and sex.

### Diagnostic methods

#### Liver necropsy

Liver necropsy was carried out in order to assign each liver a fibrosis score as well as to count the number of flukes present. In order to assign a fibrosis score in a manner that replicated that of meat inspectors on the line, fresh incisions were made parallel to and approximately 1 cm away from the meat inspector’s original cuts. Fibrosis scores of 0 to 3 were assigned with score 0 referring to a liver with no signs of fibrosis, 1 to a liver with mild focal fibrosis, 2 to a liver with severe local or mild generalised fibrosis and 3 to a liver with severe local fibrosis and calcified bile ducts or severe generalised fibrosis.

In order to count the number of parasites present the liver was cut into 1 to 2 cm slices and soaked in hot water. Flukes where collected throughout the process by squeezing each slice. The procedure was adapted from Clery *et al*.^49^ and De Bont *et al*.^50^ and the full description is included in Mazeri *et al*.^27^.

#### Faecal egg counts (FEC)

A quantitative sedimentation technique was performed as described by Sargison *et al*.^51^ to estimate the number of *F. hepatica* eggs per gram in the faecal samples collected. These were clearly differentiated from rumen fluke eggs based on the fact that *F. hepatica* eggs are browner due to bile staining and more regularly shaped than paramphistome eggs.

#### Copro antigen ELISA (cELISA)

A commercially available *F. hepatica* antigen ELISA kit (Bio-X Diagnostics, Belgium) was used to test faecal samples for the presence of excretory-secretory antigens, following the manufacturer’s instructions^33^. Equation (1) shows the way results were expressed using the sample and mean positive optical densities (OD).

1 $${\rm{Percent}}\,{\rm{Positive}}=\frac{{\rm{Sample}}\,{\rm{OD}}}{{\rm{Mean}}\,{\rm{positive}}\,{\rm{control}}\,{\rm{OD}}}\times 100$$

#### Serum antibody ELISA (sELISA)

An in-house excretory/secretory (ES) antibody ELISA, developed by the Liverpool School of Tropical Medicine, was used to test serum samples for the presence of antibodies against *F. hepatica*. We modified the procedure described by Salimi-Bejestani *et al*.^52^ by using 1:8000 monoclonal mouse anti-bovine IgG conjugate (AbD Serotec, Bio-Rad Laboratories Inc, Hertfordshire, UK). We modified the calculation provided by Salimi-Bejestani *et al*.^52^ to adjust for the use of a new positive control whereby the mean sample OD was based on two duplicates and the mean positive control was based on four duplicates (pers comm Diana Williams). Equation (2) shows how results were calculated.

2 $${\rm{Percent}}\,{\rm{Positive}}=\frac{{\rm{Mean}}\,{\rm{test}}\,{\rm{sample}}\,{\rm{OD}}}{{\rm{Mean}}\,{\rm{positive}}\,{\rm{control}}\,{\rm{OD}}}\times 111$$

### Statistical analysis

#### Mixed effects regression model

We used linear mixed effects regression models to estimate the difference in age of cattle at slaughter according to fluke status at an average slaughter weight, while controlling for differences due to sex, breed and farm-level variation. We have also adjusted for the variation between the two years of observation included in the study, as well as seasonal effects. While we cannot capture great year-on-year variation since our dataset only spans just over two years, the different levels of parasite challenge during these two years justified including year in the model. Similarly, the season during which the animals are sent to slaughter was included in the models as it can reflect different management strategies. In order to make the interpretation of the models more straightforward and to account for the fact that an animal of zero weight would not be meaningful, we centered the predictor variable, weight, around its mean^53^. Additionally, as more than one animal included in the model came from each producer, cattle coming from the same farm could not be assumed to be independent, hence the farm that each animal was consigned from was introduced in the model as a random effect. The models had the following general format, where *α* is the fixed intercept, *β* is the fixed effect of each covariate and *μ* is the random effect of farm (i).

$$\begin{array}{rcl}{\rm{Age}}\,{\rm{at}}\,{\rm{slaughter}} & \sim  & \alpha +{\beta }_{1}\times {\rm{weight}}{\rm{.centered}}\\  &  & +{\beta }_{2}\times {\rm{fluke}}+{\beta }_{3}\times {\rm{breed}}\\  &  & +{\beta }_{4}\times {\rm{sex}}+{\beta }_{5}\times {\rm{season}}\\  &  & +{\beta }_{6}\times {\rm{year}}+{\beta }_{7}{\rm{fluke}}\times {\rm{weight}}{\rm{.centered}}\\  &  & +{\beta }_{8}\times {\rm{breed}}\times {\rm{weight}}{\rm{.centered}}\\  &  & +{\beta }_{9}\times {\rm{sex}}\times {\rm{weight}}{\rm{.centered}}\\  &  & +{\beta }_{10}\times {\rm{season}}\times {\rm{weight}}{\rm{.centered}}\\  &  & +{\beta }_{11}\times {\rm{year}}\times {\rm{weight}}{\rm{.centered}}+{\mu }_{i}\times {\rm{farm}}\end{array}$$

Three regression models were built using three different measures of liver fluke infection. In the first, the “MHS model”, we used results of liver inspection by the MHS as a binary indicator of disease (two levels: liver rejected due to signs of liver fluke; and liver not rejected due to signs of liver fluke). This model used the entire 2 year abattoir dataset. In the second, the “fibrosis model”, we used the fibrosis score recorded during liver necropsy as a categorical indicator of severity of disease (four levels: fibrosis scores 0 to 3). For the third, the “burden model”, we used three different burden categories (0, 1 to 10, and more than 10 *F. hepatica* parasites) found in the liver during liver necropsy as a measure of liver fluke infection. The fibrosis and burden models used our second dataset, which included results of abattoir based sampling during 3 sampling periods and a subset of the routinely collected abattoir data.

The R statistical software^54^ was used for this analysis using scripted procedures analysis within R Studio (Version 0.98.1091)^55^. R package lme4^56^ was used for the regression analysis and confidence intervals for parameter estimates were computed using the bootstrapping method provided by the same package. Models were visually assessed for normality of the random effect, normality and homoscedasticity of the residuals as well as model fit using package predictmeans^57^.

#### Spearman correlation coefficient

We investigated the value of FECs and the two ELISA-based tests in quantifying *F. hepatica* infection measured by fluke burden and grade of fibrosis recorded at liver necropsy. We calculated the Spearman rank correlation coefficient between the number of recovered parasites in the liver and the serum and cELISA PP values as well as the number of eggs per gram counted in the faecal samples. Similar analysis was carried out comparing the grade of fibrosis reported for each liver during necropsy^58, 34, 22^, with the FECs and the ELISA-based tests. R package pspearman^59^ was used for this analysis and ggplot2^60^ was used to generate all plots of the aforementioned relationships.

#### Receiver operating characteristic (ROC) curves

For the purposes of this work ROC curves^61–64^, were used to evaluate the ability of the three diagnostic tests to distinguish between animals with: a) high vs. low or zero levels of fibrosis; and b) high vs. low or no fluke burden. In this study, high burden refers to a fluke burden of more than 10 parasites, a cut-off suggested as an economic threshold^22^. The R package Epi^65^ was used to compute values for the ROC curves, which were plotted using ggplot2^60^. It is important to note that while cELISA is a commercial kit with a defined cut-off, this cut-off varies per batch, hence we do not attempt to see how the cut-off suggested by the manufacturer’s protocol performs in this context.

## Electronic supplementary material

Supplementary information
