# Supplementary material for: Estimation of the impact of Fasciola hepatica infection on time taken for UK beef cattle to reach slaughter weight
Source: Sci Rep. 2017 Aug 4;7:7319. doi: 10.1038/s41598-017-07396-1 (PMC5544673; doi:10.1038/s41598-017-07396-1)
Supplement: Supplementary file 1 — Supplementary information [file 41598_2017_7396_MOESM1_ESM.pdf]

Additional information for paper titled "Estimation of the impact of *Fasciola hepatica* infection on time taken for UK beef cattle to reach slaughter weight." Authors: Stella Mazeri, Gustav Rydevik, Ian Handel, Barend M. deC. Bronsvoort and Neil Sargison

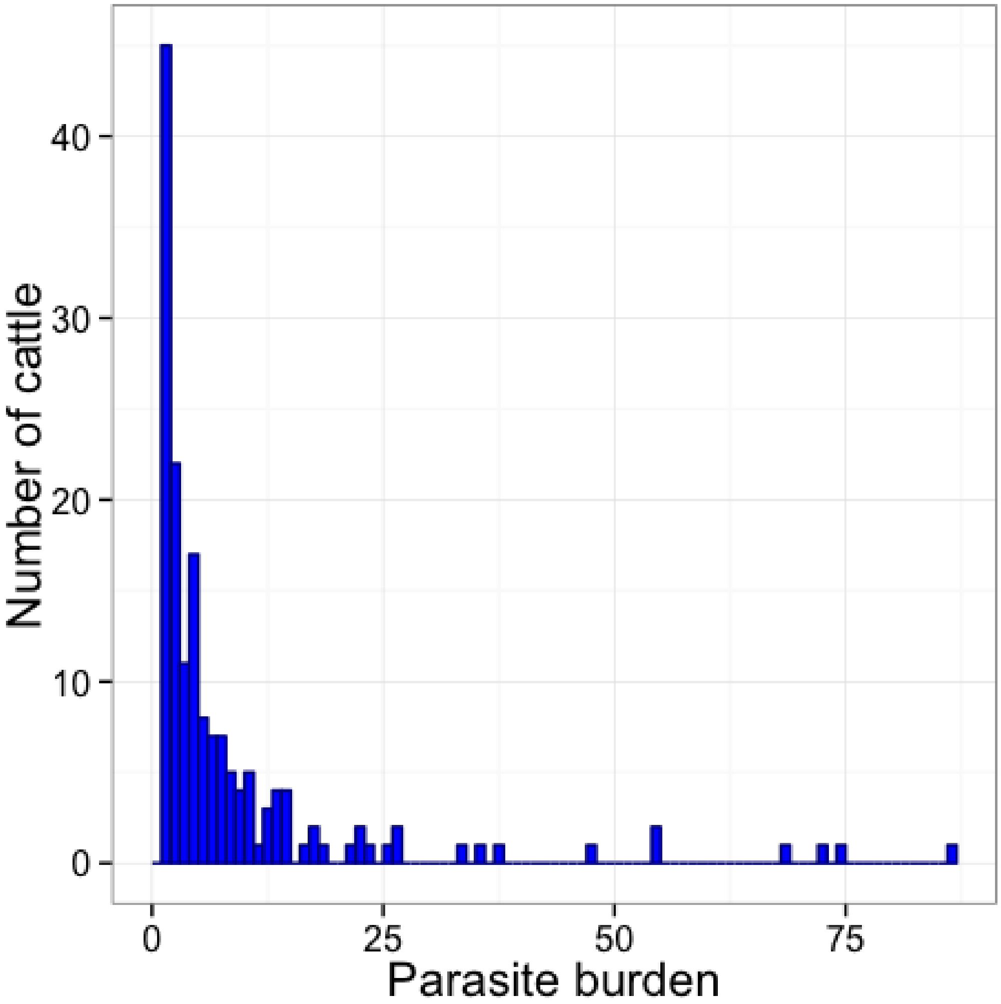

Figure S1. Distribution of parasite burden among tested cattle. Figure included animals with a parasite burden greater than 0 (n=164). The distribution is positively skewed with very few animals with burdens of more than 25 parasites.

Variable	Levels	0	1	2	3	Total
BREED	Aberdeen Angus	25	11	6	2	44
	Aberdeen Angus Cross	120	35	8	11	174
	British Blue Cross	13	1	3	1	18
	Charolais	4	7	1	0	12
	Charolais Cross	41	19	9	3	72
	Holstein Friesian	28	3	1	1	33
	Limousin	10	11	4	1	26
	Limousin Cross	68	32	12	6	118
	Other	46	22	14	3	85
	Simmental Cross	26	6	3	2	37
SEX	Female	125	56	23	11	215
	Male	241	82	32	19	374

Table S1. Distribution of levels of observed fibrosis scores, depending on breed and depending on the sex of the animal.

Table S2. MHS model results. Point estimates and confidence intervals for model parameters for estimating average difference of the age at slaughter between animals with and without fluke at the mean slaughter carcass weight of 345 kg.

Variable	Estimate	Lower 95% CI	Upper 95% CI
Intercept	741.20	736.10	746.30
fluke negative	1		
fluke positive	9.89	9.14	11.85
weight.center	0.72	0.69	0.77
sex_female	1		
sex_male	-38.91	-40.47	-37.90
breed			
Aberdeen Angus	1		
Aberdeen Angus Cross	-4.16	-6.52	-2.09
British Blue Cross	21.10	17.80	25.09
Charolais	-19.10	-26.42	-14.60
Charolais Cross	-35.14	-39.01	-33.12
Holstein Friesian	20.94	15.69	26.58
Limousin	17.30	12.33	21.29
Limousin Cross	11.07	7.92	13.81
Other	21.20	17.97	24.05
Simmental Cross	-2.87	-6.56	0.55
season_Q1	1		
season_Q2	5.09	3.06	6.10
season_Q3	1.99	0.36	3.42
season_Q4	7.08	5.89	8.72
year_2013	1		
year_2014	8.37	7.79	9.64
fluke*weight.center	-0.02	-0.03	-0.0001
sex_male*weight.center	-0.25	-0.26	-0.21
Aberdeen Angus Cross*weight.center	0.08	0.05	0.11
British Blue Cross*weight.center	0.05	0.01	0.11
Charolais*weight.center	0.15	0.08	0.28
Charolais Cross*weight.center	0.14	0.06	0.18
Holstein Friesian*weight.center	0.79	0.71	0.83
Limousin*weight.center	0.05	-0.06	0.11
Limousin Cross*weight.center	0.07	0.01	0.09
Other*weight.center	0.05	0.01	0.06
Simmental Cross*weight.center	0.02	-0.02	0.09

Table S3. Fibrosis model results. Point estimates and confidence intervals for model parameters for estimating average difference of the age at slaughter in animals with different fibrosis scores at the mean slaughter carcass weight of 345 kg.

Variable	Estimate	Lower 95% CI	Upper 95% CI
Intercept	697.20	633.70	761.60
fibrosis score 0	1	-	-
fibrosis score 1	34.02	10.58	57.42
fibrosis score 2	93.33	60.85	127.20
fibrosis score 3	78.04	32.28	124.90
weight.center	0.44	-0.41	1.33
sex_female	1		
sex_male	-41.14	-71.92	-11.32
breed			
Aberdeen Angus	1		
Aberdeen Angus Cross	-25.57	-73.45	20.91
British Blue Cross	-15.96	-85.52	45.68
Charolais	-5.45	-116.50	103.90
Charolais Cross	-43.86	-100.50	9.80
Holstein Friesian	-104.60	-193.60	-20.74
Limousin	44.89	-32.27	125.80
Limousin Cross	-27.97	-81.27	20.20
Other	-24.66	-81.63	28.09
Simmental Cross	-53.81	-112.20	2.48
season_Q1	1		
season_Q2	32.23	-21.60	85.13
season_Q3	24.54	-6.51	56.60
season_Q4	5.60	-38.50	48.98
year_2013	1		
year_2014	63.15	29.51	96.52
fibrosis score 1*weight.center	-0.04	-0.50	0.39
fibrosis score 2*weight.center	-0.23	-0.88	0.43
fibrosis score 3*weight.center	0.27	-1.05	1.54
sex_male*weight.center	-0.21	-0.77	0.31
Aberdeen Angus Cross*weight.center	0.14	-0.74	1.04
British Blue Cross*weight.center	-0.52	-2.01	1.06
Charolais*weight.center	0.36	-3.19	4.19
Charolais Cross*weight.center	0.27	-0.67	1.24
Holstein Friesian*weight.center	0.91	-0.49	2.36
Limousin*weight.center	-0.11	-1.49	1.27
Limousin Cross*weight.center	0.62	-0.24	1.55
Other*weight.center	0.34	-0.62	1.30
Simmental Cross*weight.center	0.41	-0.72	1.58

Table S4. Burden model results. Point estimates and confidence intervals for model parameters for estimating average difference of the age at slaughter in animals with different levels of burden at the mean slaughter carcass weight of 345 kg.

Variable	Estimate	Lower 95% CI	Upper 95% CI
Intercept	709.30	643.50	777.60
burden 0	1		
burden 1 to 10	31.27	5.77	56.05
burden >10	76.86	31.05	123.90
weight.center	0.38	-0.44	1.23
sex_female	1		
sex_male	-42.26	-71.95	-13.06
breed			
Aberdeen Angus	1		
Aberdeen Angus Cross	-36.37	-84.22	10.19
British Blue Cross	-27.21	-92.88	41.32
Charolais	4.82	-109.50	118.10
Charolais Cross	-49.96	-102.50	6.20
Holstein Friesian	-124.00	-211.30	-33.73
Limousin	42.09	-40.35	124.50
Limousin Cross	-38.07	-89.33	13.98
Other	-29.73	-83.80	25.46
Simmental Cross	-70.14	-126.60	-7.84
season_Q1	1		
season_Q2	41.77	-10.55	93.30
season_Q3	31.36	-0.97	61.96
season_Q4	9.64	-33.91	55.04
year_2013	1		
year_2014	68.56	34.36	103.30
burden 1 to 10*weight.center	-0.32	-0.77	0.11
burden >10*weight.center	0.93	-0.01	1.89
sex_male*weight.center	-0.20	-0.79	0.33
Aberdeen Angus Cross*weight.center	0.22	-0.65	1.06
British Blue Cross*weight.center	-0.33	-1.87	1.28
Charolais*weight.center	0.21	-3.53	3.97
CharolaisCross*weight.center	0.36	-0.61	1.29
HolsteinFriesian*weight.center	0.76	-0.67	2.18
Limousin*weight.center	0.02	-1.31	1.39
LimousinCross*weight.center	0.69	-0.22	1.58
Other*weight.center	0.35	-0.62	1.30
Simmental Cross*weight.center	0.34	-0.88	1.50
